# Construction and validation of a nomogram model for cognitive impairment in heart failure patients

**DOI:** 10.3389/fcvm.2025.1612027

**Published:** 2025-06-30

**Authors:** Ni Chen, Jie Liu, Wenjia Liu, Suzhi Zhang, Xiaolin Zhang, Bin Zhao

**Affiliations:** ^1^ Office of Academic Research, The Second Hospital of Hebei Medical University, Shijiazhuang, Hebei, China; ^2^ Department of Nursing, The Second Hospital of Hebei Medical University, Shijiazhuang, Hebei, China; ^3^ Department of Epidemiology and Health Statistics, Hebei Medical University, Shijiazhuang, Hebei, China

**Keywords:** heart failure, cognitive impairment, nomogram model, risk factors, screen tool

## Abstract

**Background:**

Patients with heart failure face a significantly elevated risk of cognitive impairment, yet clinical recognition remains inadequate—particularly among younger individuals and those with mild symptoms, leading to frequent underdiagnosis. The increasing prevalence among younger patients further worsens prognosis. This study aims to develop a tool to aid clinicians in the early identification of high-risk individuals and support informed clinical decision-making.

**Methods:**

Based on evidence-based literature and biopsychosocial holistic model of cardiovascular health, this study included 320 patients with heart failure hospitalized in the Second Hospital of Hebei Medical University from October 2023 to April 2024 to construct the model, and 80 patients from May to July 2024 were selected for temporal validation. MoCA was used to evaluate cognitive function. LASSO regression was used to select variables, Logistic regression was used to construct a nomogram model, and Bootstrap method (1,000 times) was used to evaluate the discrimination, calibration and clinical applicability of the model.

**Results:**

The incidence of cognitive impairment was 68.75% in the model group and 56.25% in the validation group. Finally, five variables including age, education level, coronary heart disease, cardiac diastolic function and physical frailty were included. The AUC of internal and temporal validation of the model were 80.2% and 72.44%, respectively, which had good prediction performance.

**Conclusion:**

The calibration curve and decision curve of the model showed a high degree of fit, which had strong clinical practicability. This model provides a reliable tool for early identification of cognitive impairment in patients with heart failure.

## 1 Introduction

Heart failure (HF) is a chronic progressive condition, with rising prevalence, readmission, and mortality rates posing a growing burden on public health systems and patients' families (1–4). With improved survival, cognitive impairment (CI) has become a common but often underrecognized complication. Studies show that HF significantly increases the risk of cognitive dysfunction; the risk of dementia is 1.52 times higher than in the general population, with an overall increase of approximately 60% (5, 6). CI affects 24%–75% of HF patients and up to 78% during acute decompensation (5, 7, 8).

Despite its high prevalence, CI is frequently missed in clinical settings. In China, the undiagnosed rate reaches 81%, with nearly half of cases overlooked-particularly in younger patients and those with mild symptoms (9). A trend toward earlier onset has been observed, likely driven by early-life cardiovascular risk factors such as hypertension, diabetes, and obesity (10).

CI impairs memory, attention, language, and executive function, reducing patients' ability to manage their condition. This leads to lower quality of life and increased risks of readmission and mortality (7, 11–13). Given its clinical impact and frequent underdiagnosis, especially among younger patients, early identification of high-risk individuals is essential. This study aims to support personalized intervention by developing a predictive tool for early risk stratification.

The Holistic Model of Cardiovascular Health, also known as the Biopsychosocial Holistic Model, was developed by Thomas et al. (14) based on Enge's Biopsychosocial Model (15). It emphasizes the interplay of physiological, psychological, behavioral, and social-environmental factors in shaping cardiovascular health. By integrating various factors, nomogram model can estimate an individual's likelihood of developing a condition or experiencing symptoms, facilitating early prevention and screening. However, existing HF prediction models have significant limitations in factor selection and model construction. Few studies have reported on systematic factor screening methods (16, 17), and over 80% of models exhibit a high risk of bias. Notably, only 14% have undergone external validation (18).

Therefore, grounded in the biopsychosocial holistic model of cardiovascular health, this study employed evidence-based methods to identify predictive factors for CI in HF patients. Predictive variables were screened using Least Absolute Shrinkage and Selection Operator (LASSO) regression, and a nomogram model was constructed via logistic regression. The model was subsequently validated and evaluated to provide a reliable tool for early CI detection in HF patients, supporting the development of personalized intervention strategies.

## 2 Materials and methods

### 2.1 Study subjects and grouping

This study recruited patients diagnosed with HF from the Department of Cardiology at a tertiary hospital in Hebei Province, China. Data collection and cognitive function assessments were conducted on these patients. Those hospitalized between October 2023 and April 2024 were designated as the modeling group, while those hospitalized from May to July 2024 formed the temporal (external) validation group.

Inclusion criteria: (1) diagnosis of HF in accordance with the *Guidelines for the Diagnosis and Treatment of Heart Failure in China (2018)* (19); (2) age ≥18 years; (3) hospitalized for >24 h in stable condition; (4) provided informed consent along with their family members.

Exclusion criteria: (1) patients with significant aphasia, hearing impairment, or inability to cooperate; (2) patients with severe systemic diseases, such as malignant tumors or mental illnesses.

Informed consent was obtained from each patient, and the study protocol was in accordance with the ethical guidelines of the 1975 Declaration of Helsinki. The study was approved by the Ethics Committee of the Second Hospital of Hebei Medical University before the start of the study (Approval No.: 2023-R563).

### 2.2 Sample size calculation

This study employed LASSO regression for predictor selection. According to the rule of thumb requiring at least 10 events per variable, and with 32 predictors included, a minimum of 320 participants were required for the modeling group. For temporal validation the recommended sample size is typically 1/4–1/2 that of the modeling group (20). In this study, 1/4 was adopted, resulting in 80 participants in the validation group.

### 2.3 Methods

Researchers conducted a comprehensive search across 11 Chinese and English databases, including PubMed, Web of Science, CINAHL, The Cochrane Library, OpenGrey, WorldWideScience, CNKI, VIP, Wanfang, China Biomedical Literature Database, and CNKI Dissertations, covering all records up to July 14, 2023. Observational studies on factors influencing CI in HF patients were screened. Following rigorous quality assessment and data extraction, 30 studies were included, identifying 32 CI risk factors across physiological, psychological, and social dimensions.

#### 2.3.1 Cognitive function assessment

Cognitive function was assessed using the Chinese version of the Montreal Cognitive Assessment (MoCA), originally developed by Nasreddine et al. (21) and subsequently translated and validated by Wang et al. (22). The MoCA is widely used for cognitive screening in older adults and patients with chronic diseases in China, and has demonstrated superior sensitivity and validity compared to the Mini-Mental State Examination (MMSE) (23).

The MoCA assesses eight cognitive domains: visuospatial, executive function, naming, memory, attention, language, abstraction, delayed recall, and orientation. It consists of 11 items with a total score of 30. A score below 26 indicates CI, according to the original criteria. To control for the effect of education level, one point was added to the total score for participants with 12 or fewer years of formal education, as recommended in the official MoCA guidelines.

#### 2.3.2 Clinical data collection

(1) On-site Questionnaire Survey: Gender (male, female), age (18–59, 60–74, ≥75), smoking status (yes/no), insomnia (yes/no), sleep duration (<6 h, 6–8 h, >8 h), marital status (with spouse/without spouse), Residence (alone/with family), and educational level (Primary education: primary school or below, Secondary education: junior/senior high school, Higher education: college or above).
(2) Electronic Medical Records: Systolic blood pressure: SBP (<90 mm Hg, 90–139 mm Hg, ≥140 mm Hg), diastolic blood pressure: DBP (<60 mm Hg, 60–89 mm Hg, ≥90 mm Hg), NYHA Functional Classification (II, III, IV), hypertension (yes/no), Diabetes (yes/no), coronary heart disease: CHD (yes/no), atrial fibrillation: AF (yes/no), ischemic cardiomyopathy: ICM (yes/no), stroke (yes/no), chronic obstructive pulmonary disease: COPD (yes/no), and renal dysfunction (yes/no).
(3) Echocardiographic Parameters: Left ventricular ejection fraction: LVEF (<55%, 55%–65%, >65%), ventricular wall motion (normal/abnormal), and diastolic function: EA (grade 0, I, II, III).
(4) Laboratory Tests: LDL cholesterol: LDL-C (<2.07 mmol/L, 2.07–3.37 mmol/L, >3.37 mmol/L), hemoglobin:HGB (<130 g/L, 130–175 g/L, >175 g/L), NT-proBNP (specific value), and high-sensitivity C-reactive protein: hs-CRP (specific value).
(5) Scale assessment tool:
  a. Medication Adherence: Assessed using the 12-item Medication Adherence Scale (MAS) (24); total score = 60. Higher scores indicate better adherence.
  b. Physical Frailty: Evaluated by the FRAIL Scale (25); total score = 5. Higher scores reflect greater frailty.
  c. Anxiety and Depression: Measured using the Hospital Anxiety and Depression Scale (HADS) (26); odd-numbered items for anxiety, even-numbered items for depression. Higher scores indicate greater severity.
  d. Quality of Life: Assessed with the Minnesota Living with Heart Failure Questionnaire (MLHFQ) (27); total score = 105. Higher scores indicate lower quality of life.
  e. Social Support: Measured by the Social Support Rating Scale (SSRS) (28); total score = 40. Higher scores indicate stronger social support.

### 2.4 Statistical analysis

Statistical analyses were conducted using SPSS 27.0 and R 4.4.1.

#### 2.4.1 Handling of missing values

Missing data in this study primarily involved examination and laboratory variables and were assumed to be missing at random (MAR), with relatively low missing rates. Median imputation was applied to LDL-C, HGB, NT-proBNP, hs-CRP, and LVEF using SPSS software. Ventricular wall motion and EA were imputed using multiple imputation methods.

#### 2.4.2 Statistical description

The incidence of CI in HF patients in both the modeling and temporal validation groups was analyzed using SPSS.

#### 2.4.3 Preliminary screening of predictive variables

To prevent overfitting and multicollinearity, LASSO regression was used for preliminary variable selection. By incorporating L1 regularization, LASSO automatically identifies key features while eliminating irrelevant variables, enhancing model generalizability and interpretability. The variable selection process was implemented using the “glmnet” package in R, with cross-validation error and coefficient path plots generated to visualize the screening process.

#### 2.4.4 Construction of the nomogram model

First, the “car” package in R was used to assess collinearity among LASSO-selected variables by calculating the variance inflation factor (VIF) and tolerance. The bootstrap method was applied with 1,000 resamples to assess the stability of the predictors, and the selection frequency of each variable was reported. After confirming that each variable is stable, logistic regression analysis was performed using the “MASS” package. The final model variables were determined through stepwise regression, and regression coefficients (B), Standard Error (SE), Wald values, odds ratios (OR), 95% confidence intervals (95% CI), and *P*-values were calculated. A significance level of *P* < 0.05 was considered statistically significant. The nomogram-based risk nomogram model was constructed using the “rms” package in R.

#### 2.4.5 Model validation and evaluation

The model was internally validated using 1,000 bootstrap resampling iterations and externally validated via temporal validation. The model's performance was assessed based on discrimination, calibration, and clinical utility:

(1) Discrimination: As the outcome variable is binary, receiver operating characteristic (ROC) analysis was used to evaluate discrimination. The ROC curve was generated using the “PROC” package in R.
(2) Calibration: Model calibration was assessed using a calibration curve, plotted with the “rms” package in R.
(3) Clinical Utility: Decision curve analysis (DCA) was performed using the “rmda” package in R to evaluate the model's clinical applicability.

## 3 Results

### 3.1 Incidence of CI in patients with HF

The incidence of CI in HF patients was 68.75% (220/320) in the modeling group and 56.25% (45/80) in the temporal validation group.

### 3.2 Screening of predictive factors for CI in HF patients

#### 3.2.1 Preliminary screening of predictive variables by LASSO regression

**Figure 1:**
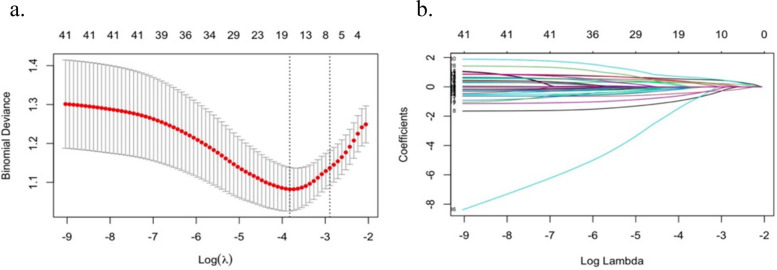
Preliminary screening of predictor variables by LASSO regression **(a)** cross-validation error plot; **(b)** coefficient path plot.

CI in HF patients was set as the outcome variable, and 32 independent variables were initially included. LASSO regression was applied for variable selection. The screening process generated a cross-validation error plot and a coefficient path plot, as shown in Figure 1. Figure 1a is a cross-validation error plot, which evaluates the impact of different penalty parameter (*λ*) values on the model using 10-fold cross-validation. Figure 1b is a coefficient path plot, illustrating the changes in regression coefficients of each variable under varying *λ* values. As *λ* increases, LASSO regression gradually shrinks the coefficients of less relevant variables to zero, thereby optimizing variable selection.

Through cross-validation, the optimal *λ* value was determined as 0.022, resulting in the selection of 15 key predictor variables, including: Gender, age, education level, smoking, sleep duration, DBP, NYHA heart function class, CHD, ICM, LVEF, EA, LDL-C, HGB, medication adherence, physical frailty.

#### 3.2.2 Stability assessment of variables selected by LASSO regression

Collinearity analysis of the 15 variables selected by LASSO regression showed tolerance >0.1 and VIF <10, indicating no significant collinearity (Table 1). Thus, a multivariate logistic regression model could be constructed.

**Table 1:** Collinearity diagnostic results of selected variables by LASSO regression.

Variables	Tolerance	VIF
Gender	0.615	1.626
Age	0.673	1.486
Education level	0.853	0.172
Smoking	0.628	1.592
Sleep duration	0.945	1.508
DBP	0.920	1.087
NYHA heart function class	0.831	1.204
CHD	0.756	1.322
ICM	0.864	1.157
LVEF	0.689	1.452
EA	0.728	1.374
LDL-C	0.905	1.104
HGB	0.855	1.170
Medication adherence	0.866	1.155
Physical frailty	0.795	1.257

**Figure 2:**
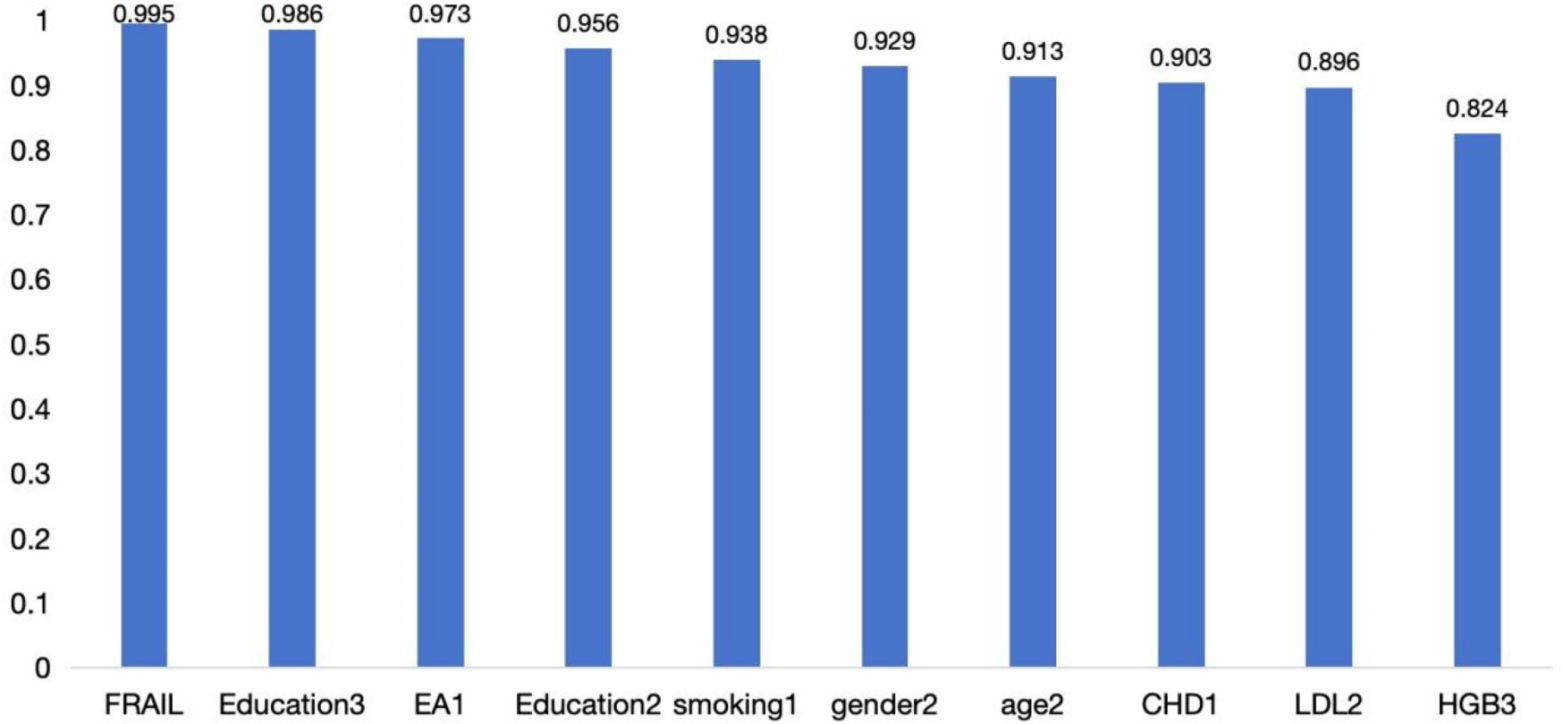
Top 10 variables by selection frequency in LASSO bootstrap analysis.

In addition, the bootstrap stability analysis showed that most core variables were selected in over 80% of the resamples, indicating high stability across different sampling scenarios (Figure 2).

#### 3.2.3 Multivariate logistic regression analysis to determine predictive variables

Binary logistic regression of the 15 LASSO-selected variables (Table 2) identified age, education level, CHD, cardiac diastolic function (EA), and physical frailty as significant predictors of CI in HF patients (Table 3).

**Table 2:** Assignment table for LASSO-logistic regression analysis of CI in HF patients.

Variables	Variable assignment
Gender	1 = “male”, 2 = “female”
Age	1 = “18–59”, 2 = “60–74”, 3 = “≥75”
Education level	1 = “Primary education”, 2 = “Secondary education”, 3 = “Higher education”
Smoking	0 = “not smoking”, 1 = “smoking”
Sleep duration	1 = “<6 h”, 2 = “6–8 h”, 3 = “>8 h”
DBP	1 = “<60 mmHg”, 2 = “60–89 mmHg”, 3 = “≥90 mmHg”
NYHA heart function class	2 = “Level II”, 3 = “Level III”, 4 = “Level IV”
CHD	0 = “No”, 1 = “Yes”
ICM	0 = “No”, 1 = “Yes”
LVEF	1 = “<55%”, 2 = “55%–65%”, 3 = “>65%”
EA	0 = “0”, 1 = “I”, 2 = “II”, 3 = “III”
LDL-C	1 = “<2.07 mmol/L”, 2 = “2.07–3.37 mmol/L”, 3 = “>3.37 mmol/L”
HGB	1 = “<130 g/L”, 2 = “130–175 g/L”, 3 = “>175 g/L”
Medication adherence	Entering numeric variables
Physical frailty	Entering numeric variables
MoCA	0 = “No CI”, 1 = “CI”

**Table 3:** The LASSO-logistic regression analysis results of CI in HF.

Variables	Grouping	*B*	*SE*	*Wald*	*P*	*OR*	95%CI
Intercept		0.340	1.351	0.252	0.801	1.405	[0.100, 19.831]
Age	18–59	0^a^					
	60–74	0.695	0.323	2.152	0.031	2.004	[1.064, 3.774]
	≥75	0.658	0.550	1.196	0.232	1.931	[0.657, 5.677]
Education level	Primary education	0^a^					
	Secondary education	−1.093	0.407	−2.687	0.007	0.335	[0.151, 0.744]
	Higher education	−1.540	0.473	−3.258	0.001	0.214	[0.085, 0.541]
	Yes	0.777	0.342	2.271	0.023	2.174	[1.112, 4.250]
Coronary heart disease	No	0^a^					
Cardiac diastolic function (EA)	0	0^a^					
	I	1.770	0.645	2.745	0.006	5.869	[1.659, 20.769]
	II	0.988	0.610	1.630	0.103	2.686	[0.819, 8.809]
	III	1.000	0.820	1.220	0.223	2.719	[0.545, 13.566]
Physical frailty		0.371	0.130	2.853	0.004	1.450	[1.123, 1.871]

^a^ 0 means this group is the reference group.

### 3.3 Construction and validation of a nomogram model for CI in HF patients

#### 3.3.1 Construction of a nomogram model

**Figure 3:**
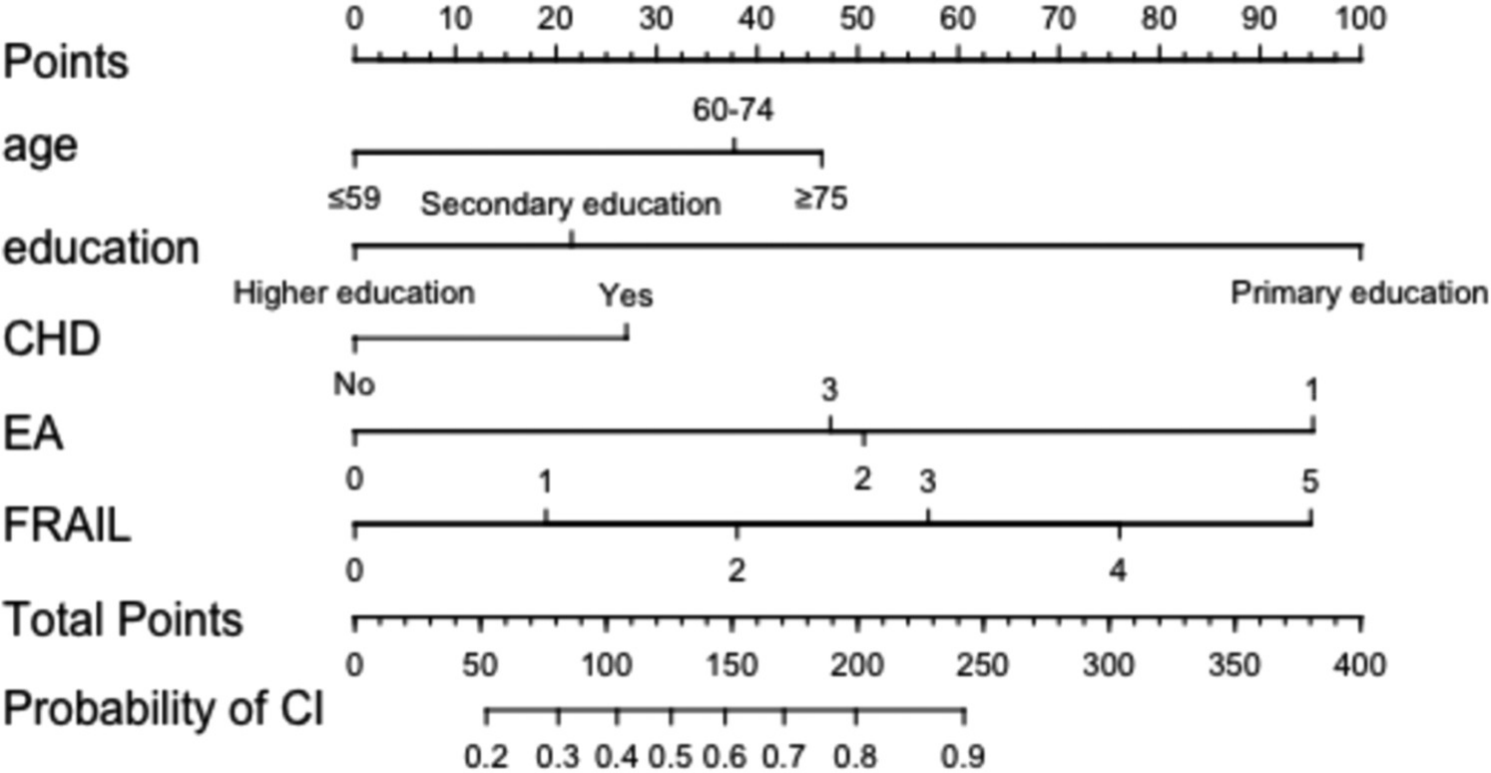
Nomogram model for developing CI in HF patients. CHD, coronary heart disease; EA, cardiac diastolic function was measured by echocardiography.

The five significant independent variables identified in multivariate logistic regression—age, education level, CHD, cardiac diastolic function (EA), and physical frailty—were used to develop a nomogram model for CI in HF patients using R software. The nomogram visually represents the contribution of each predictor by assigning scores to variables and summing them to estimate the probability of CI in HF patients (See Figure 3).

#### 3.3.2 Internal validation

**Figure 4:**
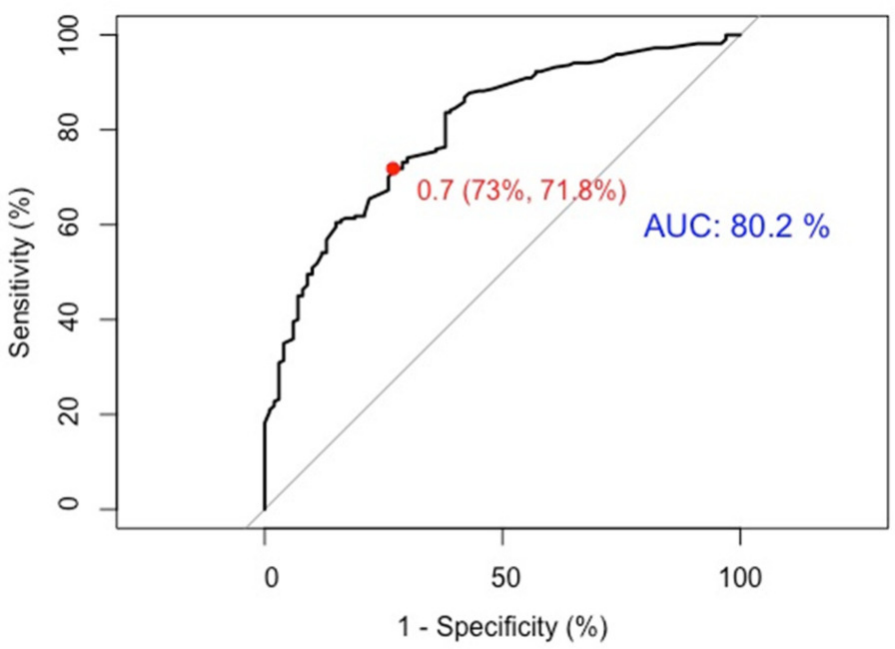
ROC curve for internal validation of CI nomogram model in HF patients.

**Figure 5:**
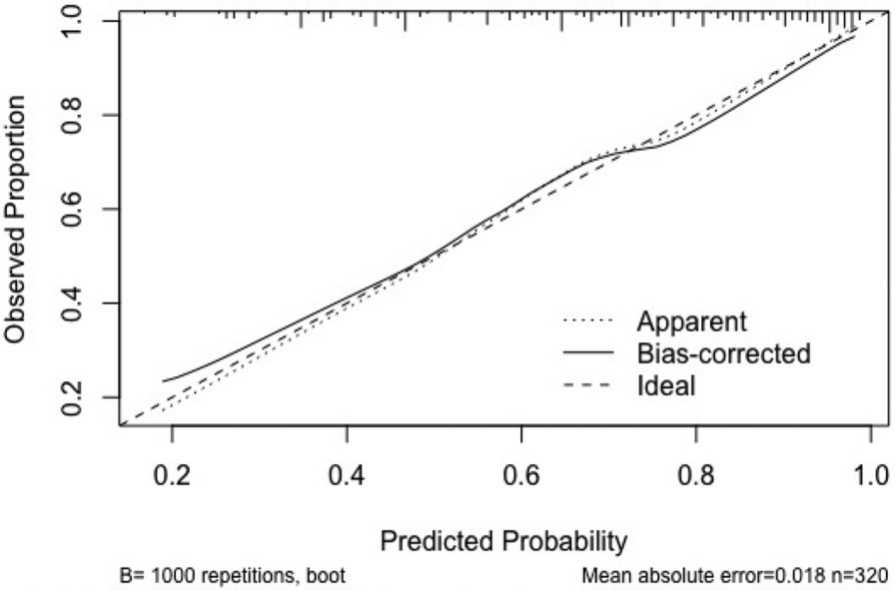
Calibration curves for internal validation of CI nomogram model in HF patients.

**Figure 6:**
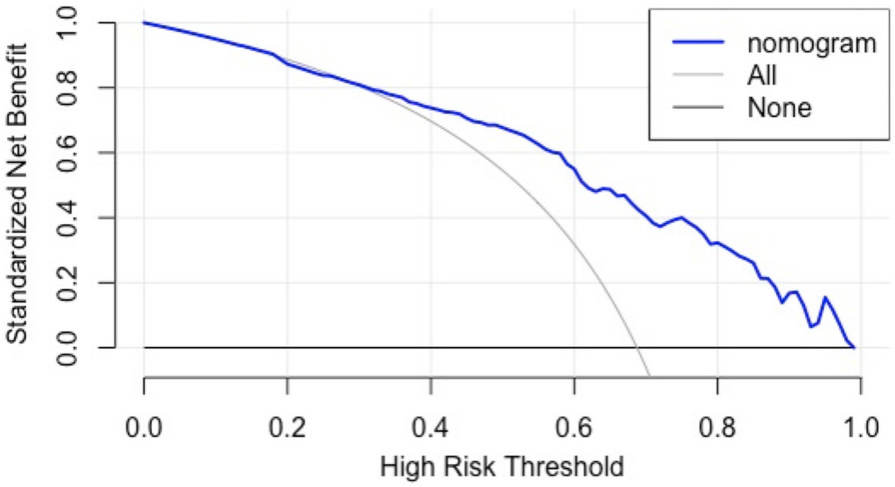
DCA curves for internal validation of CI nomogram model in HF patients.

(1) Discrimination: The model was internally validated using 1,000 Bootstrap cycles. The optimal ROC cutoff was 0.7, with a sensitivity of 71.8% and specificity of 73%. The AUC was 80.2%, indicating good discrimination (See Figure 4).
(2) Calibration: The Apparent curve and Bias-corrected curve in the calibration plot closely align with the Ideal curve, indicating good model fit, predictive ability, and calibration (See Figure 5).
(3) Clinical utility: The DCA curve shows that for threshold probabilities >0.2, the blue curve is notably higher than the “All” (gray) and “None” (black) curves, indicating significant clinical net benefit across most threshold ranges (See Figure 6).

#### 3.3.3 Comparison of baseline characteristics between the modeling group and the temporal validation group

There were no statistically significant differences in most variables between the two groups (*P* > 0.05), indicating comparability in overall demographic and clinical characteristics. However, significant differences were observed in insomnia status (*P* = 0.049), sleep duration (*P* < 0.001), frailty score (*P* < 0.001), EA (*P* < 0.001), hs-CRP (*P* = 0.023), and depression score (*P* < 0.001) (See Table 4).

**Table 4:** Comparison table of baseline characteristics between modeling group and temporal validation group.

Variables	Modeling group (*n* = 320)	Temporal validation group (*n* = 80)	*χ*^2^/*t*/*Z*	*P*
Gender [*n* (%)]			0.003^a^	0.958
Male	207 (64.70%)	52 (65%)		
Female	113 (35.30%)	28 (35%)		
Age(year) [*n* (%)]			1.470^a^	0.480
18∼	108 (33.80%)	28 (35%)		
60∼	160 (50%)	35 (43.80%)		
75∼	52 (16.30%)	17 (21.30%)		
Smoking [*n* (%)]			0.713^a^	0.398
No	208 (65%)	56 (70%)		
Yes	112 (35%)	24 (30%)		
Insomnia [*n* (%)]			3.884^a^	0.049
No	191 (59.7%)	38 (47.5%)		
Yes	129 (40.3%)	42 (52.5%)		
Sleep duration (h) [*n* (%)]			33.245^a^	<0.001
<6	19 (5.9%)	22 (27.5%)		
6∼	208 (65%)	44 (55%)		
8∼	93 (29.1%)	14 (17.5%)		
SBP (mm Hg) [*n* (%)]			0.014^a^	0.993
<90	4 (1.3%)	1 (1.3%)		
90∼	242 (75.6%)	61 (76.3%)		
140∼	74 (23.1%)	18 (22.5%)		
DBP (mm Hg) [*n* (%)]			0.496^a^	0.780
<60	12 (3.8%)	3 (3.8%)		
60∼	228 (71.3%)	60 (75%)		
90∼	80 (25%)	17 (21.3%)		
NYHA heart function class [*n* (%)]			0.940^a^	0.625
Ⅱ	102 (31.9%)	30 (37.5%)		
III	168 (52.5%)	38 (47.5%)		
Ⅳ	50 (15.6%)	12 (15%)		
Hypertension [*n* (%)]			0.280^a^	0.597
No	110 (34.4%)	25 (31.3%)		
Yes	210 (65.6%)	55 (68.8%)		
Diabetes [*n* (%)]			0.049^a^	0.825
No	228 (71.3%)	58 (72.5%)		
Yes	92 (28.7%)	22 (27.5%)		
CHD [*n* (%)]			0.197^a^	0.657
No	92 (28.7%)	21 (26.3%)		
Yes	228 (71.3%)	59 (73.8%)		
AF [*n* (%)]			0.043^a^	0.836
No	200 (62.5%)	51 (63.7%)		
Yes	120 (37.5%)	29 (36.3%)		
ICM [*n* (%)]			0.220^a^	0.639
No	265 (82.8%)	68 (85%)		
Yes	55 (17.2%)	12 (15%)		
Stroke [*n* (%)]			0.139^a^	0.709
No	254 (79.4%)	65 (81.3%)		
Yes	66 (20.6%)	15 (18.8%)		
COPD [*n* (%)]			1.272^a^	0.259
No	308 (96.3%)	79 (98.8%)		
Yes	12 (3.8%)	1 (1.3%)		
Renal dysfunction [*n* (%)]			0.053^a^	0.817
No	281 (87.8%)	71 (88.8%)		
Yes	39 (12.2%)	9 (11.3%)		
Medication adherence [*M*(*QR*)]	44 (10)	43 (10)	−0.267^c^	0.790
physical frailty [*M*(*QR*)]	2 (2)	2 (1)	−3.553^c^	<0.001
LVEF (%) [*n* (%)]			3.016^a^	0.221
<55	234 (73.1%)	66 (82.5%)		
55∼	69 (21.6%)	11 (13.8%)		
65∼	17 (5.3%)	3 (3.8%)		
ventricular wall motion [*n* (%)]			0.000^a^	1.000
Abnormal	236 (73.8%)	59 (73.8%)		
Normal	84 (26.3%)	21 (26.3%)		
EA [*n* (%)]			62.368^a^	<0.001
0	19 (5.9%)	22 (27.5%)		
Ⅰ	106 (33.1%)	42 (52.5%)		
Ⅱ	175 (54.7%)	9 (11.3%)		
III	20 (6.3%)	7 (8.8%)		
LDL-C(mmol/L) [*n* (%)]			3.314^a^	0.191
<2.07	96 (30%)	24 (30%)		
2.07∼	171 (53.4%)	49 (61.3%)		
3.37∼	53 (16.6%)	7 (8.8%)		
HGB (g/L) [*n* (%)]			4.317^a^	0.116
<130	73 (22.8%)	24 (30%)		
130∼	245 (76.6%)	54 (67.5%)		
175∼	2 (0.6%)	2 (2.5%)		
NT-proBNP	1,170 (2,160.50)	1,090 (2,837.50)	−0.316^c^	0.752
hs-CRP	4.65 (9.16)	2.55 (5.98)	−2.274^c^	0.023
Anxiety [*M*(*QR*)]	9 (3)	8 (3)	−0.001^c^	1.000
Depression [*M*(*QR*)]	9 (3)	11 (3)	−4.744^c^	<0.001
Marital status [*n* (%)]			0.271^a^	0.603
with spouse	264 (82.5%)	64 (80%)		
without spouse	56 (17.5%)	16 (20%)		
Residence [*n* (%)]			2.996^a^	0.083
Alone	37 (11.6%)	4 (5%)		
with family	283 (88.4%)	76 (95%)		
Education level [*n* (%)]			0.164^a^	0.921
Primary education	106 (33.1%)	28 (35%)		
Secondary education	156 (48.8%)	37 (46.3%)		
Higher education	58 (18.1%)	15 (18.8%)		
MLHFQ (*χ*^2^ ± S)	46.52 ± 16.12	47.10 ± 12.14	−0.359^b^	0.720
SSRS [*M*(*QR*)]	28(5)	29(6)n	−1.718^c^	0.086
CI [*n* (%)]			4.4.72^a^	0.034
No	100(31.3%)	35(43.8%)		
Yes	220(68.8%)	45(56.3%)		

^a^ *χ*^2^ value.

^b^ *t* value.

^c^ *Z* value.

#### 3.3.4 Temporal validation

This study finally included 80 patients with HF from May to July 2024 for temporal validation of the model. The evaluation results are as follows.

**Figure 7:**
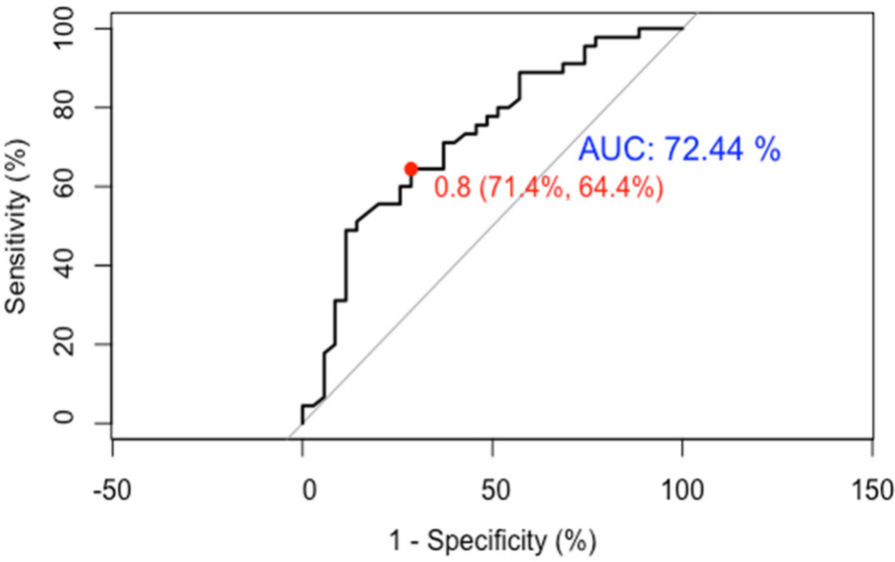
ROC curves for temporal validation of CI nomogram model in HF patients.

**Figure 8:**
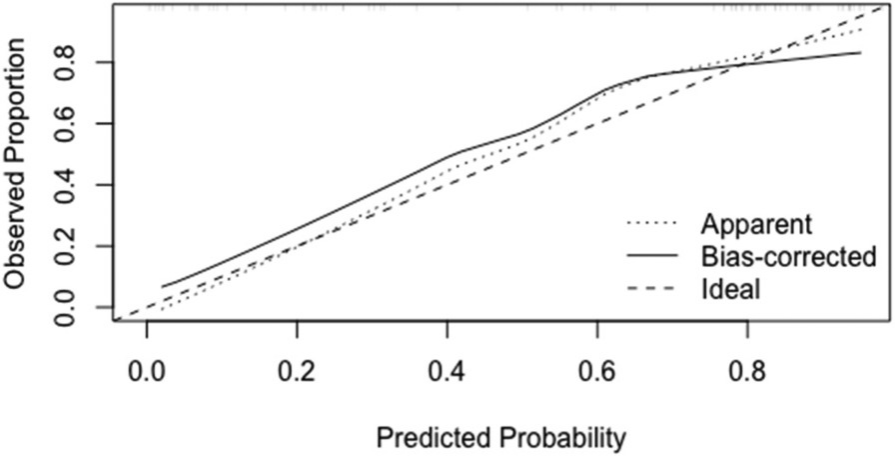
Calibration curves for temporal validation of CI nomogram model in HF patients.

**Figure 9:**
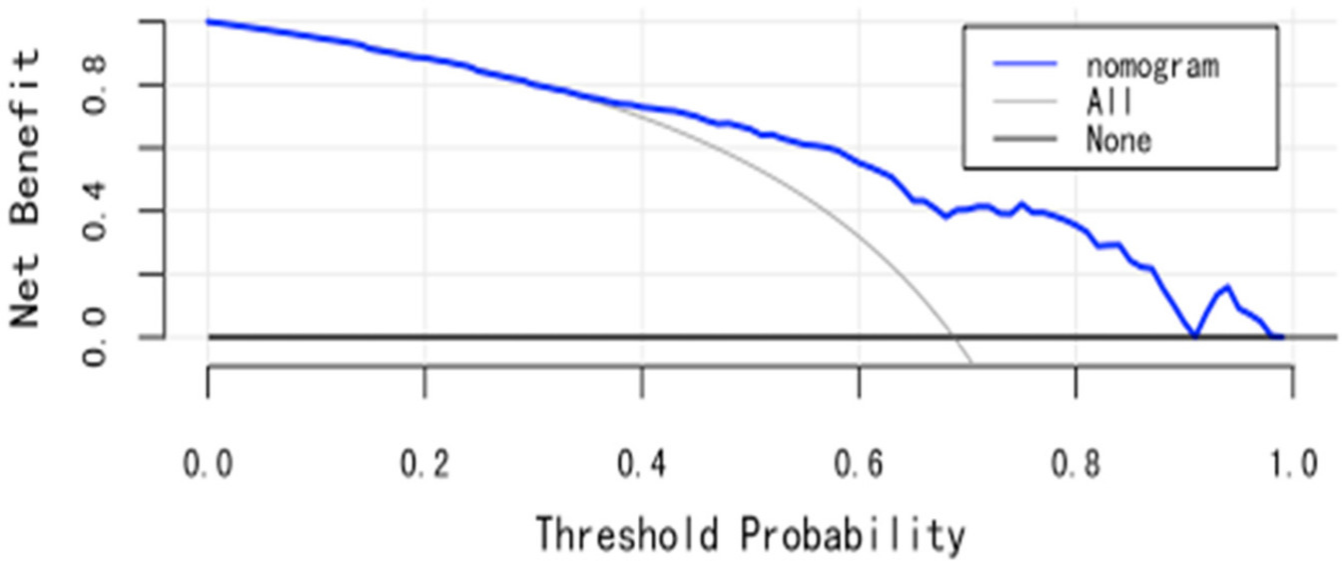
DCA curves for temporal validation of CI nomogram model in HF patients.

(1) Discrimination: Temporal validation with 80 HF patients showed an optimal ROC cutoff of 0.8, sensitivity of 64.4%, and specificity of 71.4%. The AUC was 72.44%, indicating good discrimination (See Figure 7).
(2) Calibration: In the temporal validation calibration curve, the Apparent curve and Bias-corrected curve closely align with the Ideal curve, indicating good model fit, predictive ability, and calibration (See Figure 8).
(3) Clinical utility: In temporal validation, the DCA curve shows that for threshold probabilities >0.2, the blue curve provides a significantly higher net benefit than the “All” (gray) and “None” (black) strategies (See Figure 9).

## 4 Discussion

Based on the biopsychosocial holistic model of cardiovascular health, this study systematically identified 32 risk factors for CI in HF patients using evidence-based methods. Clinical data collection and cognitive assessments were conducted to develop a nomogram model, which was internally and externally validated. The results demonstrated good discrimination, calibration, and clinical applicability, indicating strong external validity. This model provides an effective prediction tool for HF patients, offering valuable nursing guidance.

### 4.1 Discussion on key predictors of CI in HF

The final nomogram model included five key variables: age, education level, CHD, physical frailty, and cardiac diastolic function. Their impact mechanisms on cognitive function in HF patients are discussed below.

Age is a well-established risk factor for CI in HF patients (29), with studies showing a significant increase in risk, especially beyond 69 years (30). Although this study included patients ≥18 years, 66.25% were over 60, reinforcing the strong association between aging and CI, consistent with previous research (31). Education level affects cognitive function by influencing cognitive reserve and health behaviors. Higher education is linked to stronger cognitive resilience and better coping strategies, whereas lower education levels may increase cognitive decline risk, particularly in chronic diseases like HF (32). CHD can impair cognitive function through vascular damage and reduced cerebral blood flow. Conversely, CI can worsen self-management, exacerbating CHD symptoms and creating a vicious cycle (33). Cardiac diastolic dysfunction may contribute to cerebral microcirculatory impairment via reduced cardiac output, systemic microcirculatory disorders, chronic inflammation, and oxidative stress, further impacting cognition (34, 35). Physical frailty, common in HF, is linked to reduced physical activity, exacerbating cognitive decline. Studies show frailty in HF patients correlates with poorer cognitive function, lower quality of life, and increased healthcare needs (36).

In addition, potential interactions may exist among various risk factors. For example, elderly patients with low educational attainment may have reduced cognitive reserve, making them more susceptible to CI under chronic cerebral hypoperfusion caused by HF. Moreover, studies have shown that education does not mitigate age-related cognitive decline (37); rather, its influence is primarily on cognitive capacity in early life and does not slow the rate of decline in later years (38). This suggests that improving early-life environments may be more effective in preventing cognitive decline than interventions initiated in old age. Furthermore, physical frailty may amplify the cognitive impact of cardiac insufficiency and coronary artery disease. Frail individuals often have reduced physical activity, leading to further deterioration in cardiopulmonary function. Additionally, chronic inflammation, malnutrition, and muscle loss commonly associated with frailty may disrupt brain metabolism and neuroprotection, thereby accelerating cognitive decline (39).

Psychological factors were not included in the final model, which may be attributed to limitations such as the low sensitivity of measurement tools, sample gender imbalance, and interactions between psychological and other variables. Although the HADS (40)–commonly used in hospitalized populations–was applied, its sensitivity to mild symptoms is limited (41). In this study, most patients scored below the recommended cutoff, potentially underestimating the severity of anxiety and depression. Moreover, the sample was predominantly male. Prior studies suggest that men are more likely to underreport or suppress emotional distress, and self-report tools may be subject to response bias (42). Additionally, anxiety and depression may influence cognitive function indirectly through factors such as illness perception and psychological resilience (43), rather than acting as independent predictors. These factors may have reduced the likelihood of psychological variables being retained in the final model. Future research should consider using more sensitive instruments and incorporating a broader range of psychological measures to enhance the predictive accuracy of CI risk models.

Thus, comprehensive assessments of these variables can guide personalized nursing interventions, effectively reducing CI risk and improving quality of life in HF patients.

### 4.2 The nomogram model demonstrates good predictive performance

In this study, a risk nomogram model for CI in HF patients was developed using LASSO regression combined with logistic regression and visualized through a nomogram. Internal validation was performed using 1,000 bootstrap resamples, yielding an area under the ROC curve (AUC) of 0.80 and good calibration, indicating strong model performance. For temporal validation, an independent dataset from a different time period within the same hospital was used. The model achieved an AUC of 0.72, demonstrating robust predictive ability and reasonable generalizability across time.

Currently, risk prediction tools for CI in HF patients remain limited. In the TIME-CHF study, Kuipers (17) proposed a scoring model based on seven variables, which achieved an AUC of 0.71 in the development cohort but showed poor performance in external datasets (AUC = 0.56), highlighting its limited generalizability. In contrast, the nomogram developed in this study incorporated more biologically relevant predictors, such as physical frailty and cardiac diastolic function. The model demonstrated good discrimination and calibration in both internal and temporal validation, offering a more reliable tool for the early identification of CI in HF patients.

Although the model demonstrated good overall discrimination, its AUC declined from 80.2% in the development phase to 72.44% during temporal validation. To further explore this, subgroup analyses were conducted within the validation cohort. The model maintained strong performance in certain subgroups. For example, individuals aged 18–59 (AUC = 0.667), those without frailty (AUC = 0.967), with higher educational attainment (AUC = 0.716), without coronary heart disease (AUC = 0.878), and with severely impaired diastolic function (e.g., grade 3, AUC = 0.958). In contrast, predictive performance declined in subgroups with greater clinical heterogeneity or more complex conditions, such as patients aged 60–74 (AUC = 0.521) and those with coronary heart disease (AUC = 0.582). (See Supplementary Figure 1 for subgroup ROC curves.) This discrepancy in model performance may be attributed to several factors: (1) Cohort Effects: Temporal changes in patient characteristics–such as advances in diagnosis and treatment, improved health awareness, and evolving disease management strategies–may lead to differences between the development and validation cohorts. These cohort effects can shift the risk distribution of CI and reduce model performance in newer datasets (44, 45). (2) Unmeasured or Excluded Confounders: ① Key lifestyle factors–such as diet, physical activity, alcohol consumption, and smoking–are closely linked to cognitive function and vary significantly across socioeconomic groups (46). ② Social support and psychological status are also important, particularly among older adults. The exclusion of these variables may partially explain the reduced predictive accuracy observed in the 60–74 age group (47). ③ Treatment-Related Complexity: Medication adherence and treatment complexity can influence cognitive outcomes, especially in patients with chronic conditions like coronary artery disease, who often require multi-drug regimens and more intensive disease management. ④ Seasonal Variation: Some studies have suggested that cognitive function may fluctuate with seasonal changes (48), potentially affecting model performance. In this study, subgroup analysis by season revealed variation in AUC values: autumn (85.18%), winter (81.96%), spring (73.72%), and summer (72.44%) (see Supplementary Figure 2). Shijiazhuang, the study site, has a temperate monsoon climate characterized by cold, dry winters and hot, humid summers, with relatively mild conditions in spring and autumn. The observed seasonal differences may be explained by environmental influences on cardiovascular and cognitive function. In autumn and winter, lower temperatures and higher infection rates may exacerbate heart failure, aggravate cerebral hypoperfusion and inflammation, and thereby amplify CI signals—improving model detectability. In contrast, high temperatures and humidity in summer may increase cardiac compensatory burden, obscure clinical symptoms (e.g., fatigue masking cognitive complaints), and weaken the clarity of CI signals, leading to reduced model performance (49, 50).

Therefore, although the overall model performance declined, subgroup analyses revealed that it retained strong discriminative ability in specific populations. This suggests that future efforts could focus on optimizing or recalibrating the model based on high-risk subgroups to enhance its practicality and robustness across diverse clinical settings.

### 4.3 The nomogram model demonstrates good clinical utility

The model demonstrated meaningful clinical utility in both internal and temporal validations. Decision curve analysis showed that when the threshold probability exceeded 20%, the model provided greater net benefit compared to “treat-all” or “treat-none” strategies. This suggests that when clinicians are inclined to initiate interventions at a predicted risk above 20%, the model offers clear decision-making support. The threshold probability reflects the clinician's balance between the benefits and risks of intervention. When the predicted risk surpasses this threshold, further assessment or intervention is recommended–providing a useful reference point for evidence-based management under clinical uncertainty. However, the threshold is not fixed and should be adapted to specific clinical contexts. For low-risk, low-cost interventions, a lower threshold may be acceptable. Conversely, for high-risk or resource-intensive interventions, a higher threshold is warranted. Although this study suggests optimal model benefit at a 0.2 threshold, real-world application requires individualized judgment based on patient characteristics and healthcare context.

The variables included in the model—such as age, education level, CHD, diastolic function, and physical frailty—are all routinely collected clinical indicators, allowing for rapid risk assessment at admission or during follow-up. Based on the nomogram output, clinicians can identify patients at high risk of CI, which can inform initial screening strategies and guide the allocation of resources for targeted interventions, enabling individualized management. Specifically, when the predicted risk is below 20%, the net clinical benefit is relatively small. However, given the low cost and minimal risk of interventions such as health education, nutritional support, and lifestyle modification, routine preventive measures can still be justified. When the predicted risk exceeds 20%, the model shows a clear net benefit, and more tailored decisions are recommended based on patient-specific characteristics. For example, an elderly HF patient (≥68 years), with secondary education (junior high school), comorbid coronary heart disease, grade II diastolic dysfunction, and a frailty score of 3 may have an estimated 80% risk of CI. Such patients should be prioritized for further cognitive assessment and individualized intervention. To streamline clinical workflows, the nomogram can be integrated into electronic medical record systems for automated risk stratification and intelligent alerting. For instance, a prominent notification such as “High-Risk Heart Failure Patient: Cognitive Impairment Screening Recommended” could help ensure timely clinical attention at key decision points. This risk-based stratification approach not only supports more efficient allocation of healthcare resources but also enhances the early identification and management of CI.

### 4.4 Limitations and future directions

Firstly, this study developed a risk identification model based on cross-sectional data. While it enables assessment of CI risk at a specific time point, the absence of temporal sequencing among variables limits causal inference and prevents tracking of dynamic changes in cognitive risk over time. This highlights the need for future longitudinal studies incorporating time-series data to construct prognostic models with stronger predictive validity.

Secondly, external validation is essential for assessing a model's generalizability. Common approaches include temporal validation (across different time periods within the same institution), domain validation (across different subpopulations), and spatial validation (across different institutions or regions) (51, 52). This study employed temporal validation, which partially assessed model stability over time. However, the validation was limited to single-center data with a small sample size. Baseline imbalances and lack of population diversity or geographic variation may have contributed to the reduced model performance. Future studies should incorporate multi-center data to more comprehensively evaluate the model's robustness and applicability.

Finally, although LASSO regression was used for variable selection—with collinearity diagnostics and bootstrap stability analysis confirming the relative stability of predictors—the limited sample size and LASSO's inherent variable shrinkage may have excluded some weak but clinically relevant predictors, resulting in potential false negatives. Expanding the sample size and applying more flexible modeling techniques, such as random forests, could improve both the robustness and interpretability of future models.

## 5 Conclusion

The CI nomogram model for HF patients developed in this study demonstrated strong robustness and clinical applicability. It serves as an effective assessment tool for predicting CI risk, enabling medical staff to quickly identify high-risk patients, implement personalized interventions, and optimize nursing resource allocation, ultimately improving health outcomes and quality of life.

## Data Availability

The raw data supporting the conclusions of this article will be made available by the authors, without undue reservation.

## Supplementary material

Supplementary Figure 1 ROC curves of subgroup analysis of each variable of the nomogram model

Supplementary Figure 2 ROC curve of seasonal subgroup analysis.
