# Supplementary figures and images for: Construction and validation of a nomogram model for cognitive impairment in heart failure patients
Source: Front Cardiovasc Med. 2025 Jun 30;12:1612027. doi: 10.3389/fcvm.2025.1612027 (PMC12256441; doi:10.3389/fcvm.2025.1612027)

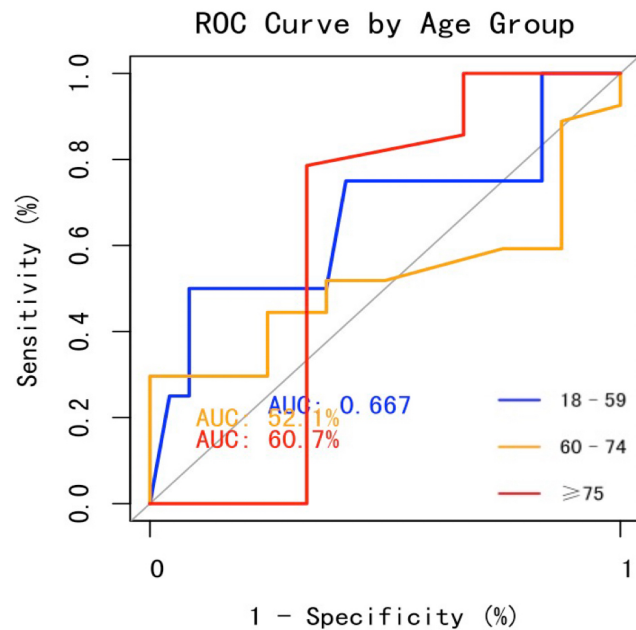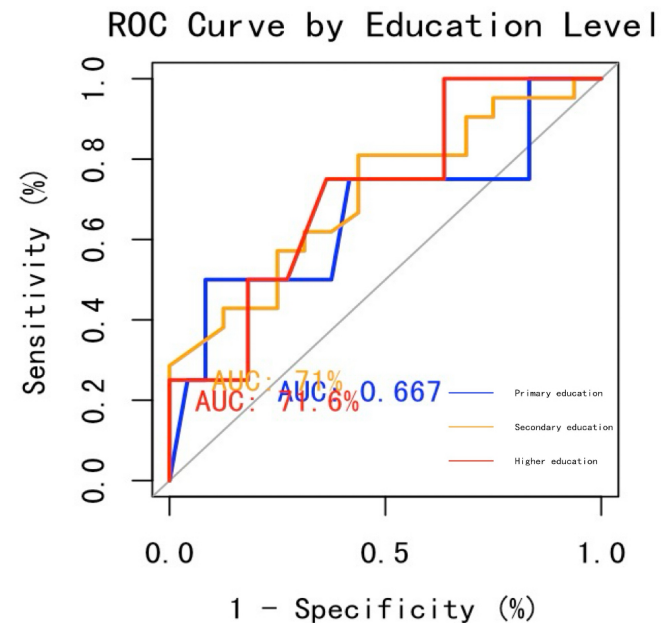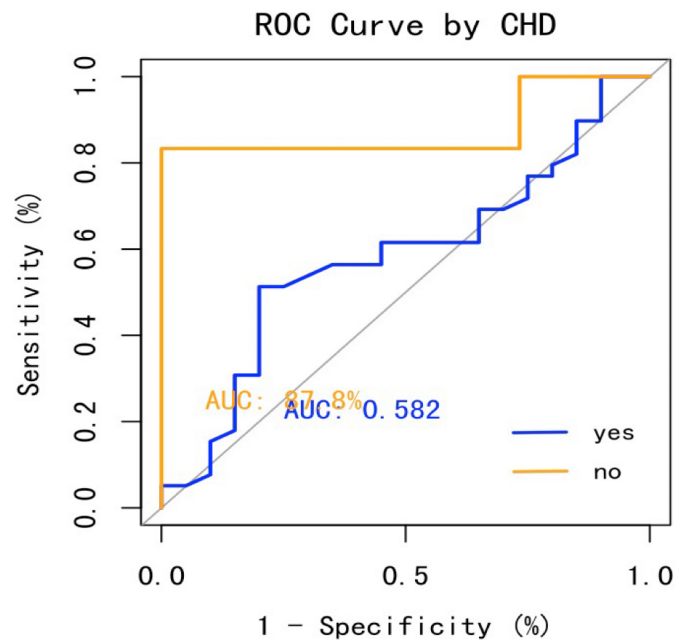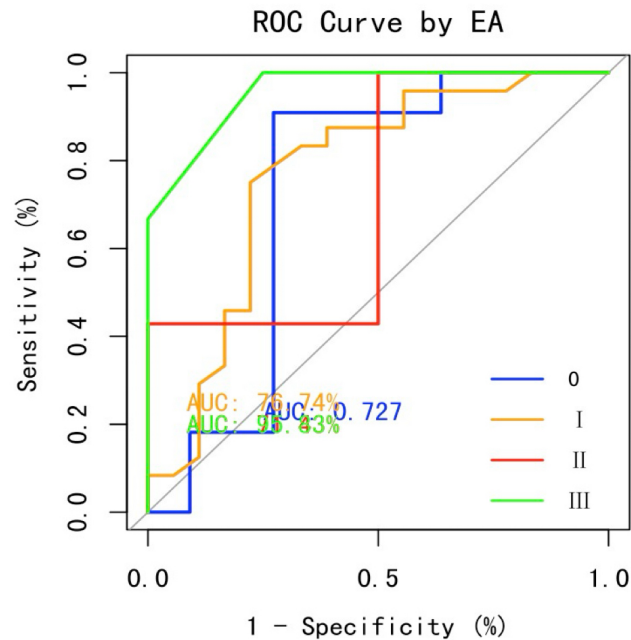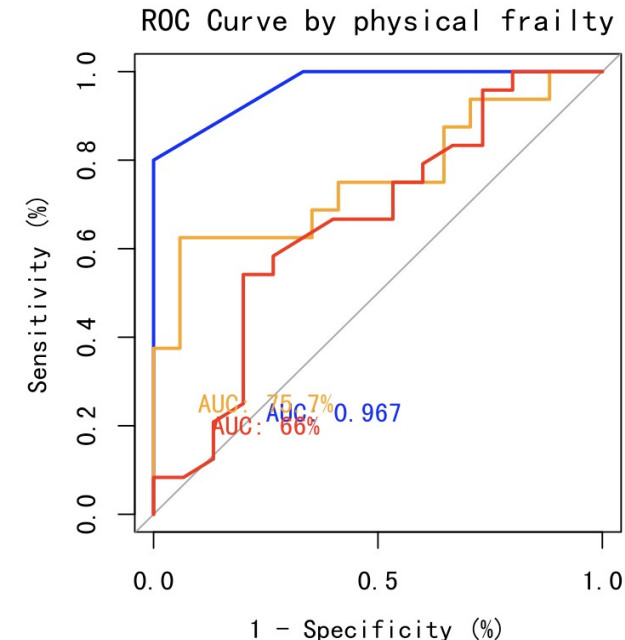

Supplement: Supplementary Figure 1 — ROC curves of subgroup analysis of each variable of the nomogram model [file Image1.pdf]

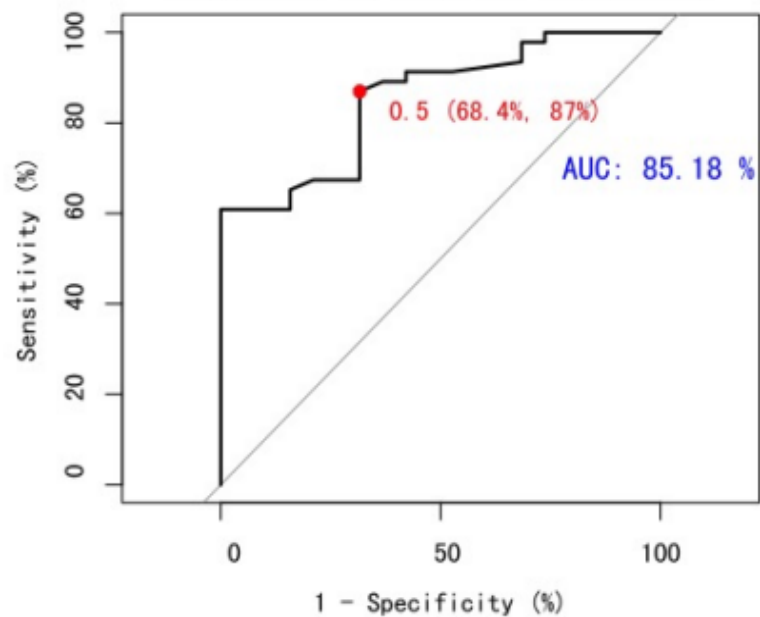

a. Autumn

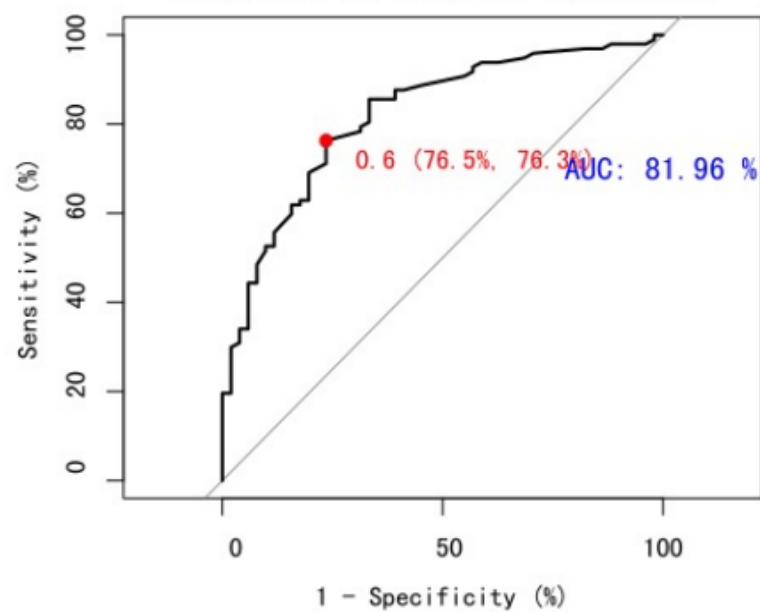

b. Winter

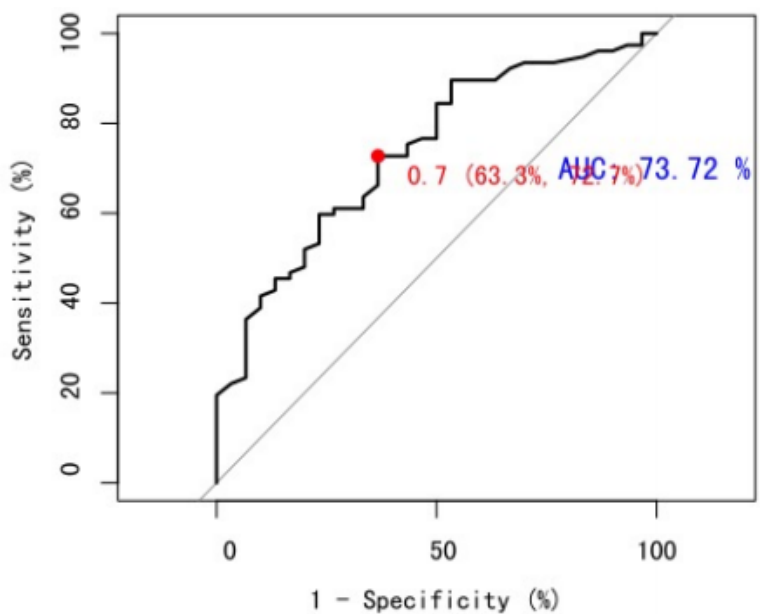

c. Spring

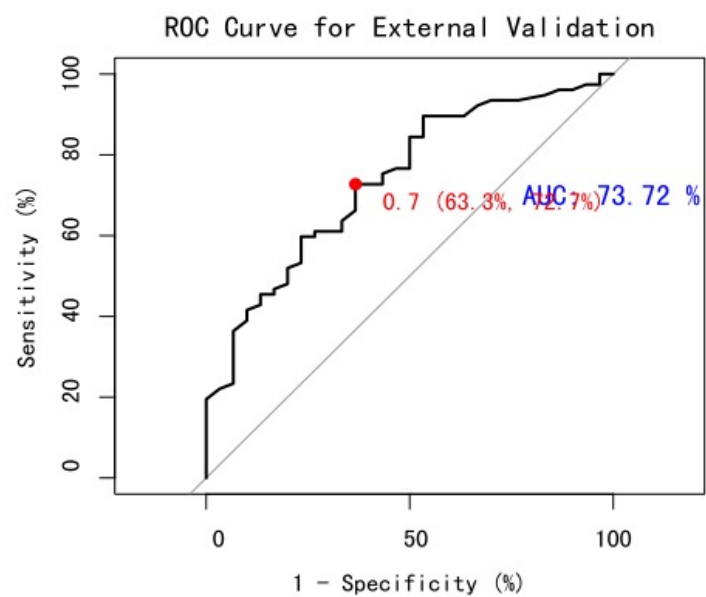

d. Summer

Supplement: Supplementary Figure 2 — ROC curve of seasonal subgroup analysis. [file Image2.pdf]
